# Trends in maternal deaths in HIV‐infected women, on a background of changing HIV management guidelines in South Africa: 1997 to 2015

**DOI:** 10.1002/jia2.25022

**Published:** 2017-11-27

**Authors:** Coceka N Mnyani, Eckhart J Buchmann, Matthew F Chersich, Karlyn A Frank, James A McIntyre

**Affiliations:** ^1^ School of Clinical Medicine Department of Obstetrics and Gynaecology University of the Witwatersrand Johannesburg South Africa; ^2^ School of Public Health University of the Witwatersrand Johannesburg South Africa; ^3^ SACEMA (DST/NRF Centre of Excellence in Epidemiological Modelling and Analysis) Stellenbosch University Stellenbosch South Africa; ^4^ Wits Reproductive Health and HIV Institute Faculty of Health Sciences University of the Witwatersrand Johannesburg South Africa; ^5^ International Centre for Reproductive Health Department of Obstetrics and Gynaecology University of Ghent Ghent Belgium; ^6^ Anova Health Institute Johannesburg South Africa; ^7^ School of Public Health and Family Medicine University of Cape Town Cape Town South Africa

**Keywords:** maternal mortality, HIV‐infected pregnant women, antiretroviral therapy, HIV treatment guidelines, South Africa, high HIV prevalence

## Abstract

**Introduction:**

As work begins towards the Sustainable Development Goal target of reducing the global maternal mortality ratio (MMR) to less than 70 deaths per 100,000 live births by 2030, much needs to be done in ending preventable maternal deaths. After 1990, South Africa experienced a reversal of gains in decreasing maternal mortality, with an increase in HIV‐related maternal deaths. In this study, we assessed trends in maternal mortality in HIV‐infected women, on a background of an evolving HIV care programme.

**Methods:**

This was a cross‐sectional, retrospective record review of maternal deaths in the obstetrics unit at Chris Hani Baragwanath Academic Hospital, in Johannesburg, South Africa, a referral hospital in a high HIV prevalence setting where the prevalence among pregnant women has plateaued around 29.0% for the past decade. Trends in HIV diagnosis and management in pregnancy, and causes of maternal deaths in HIV‐infected women were analysed over different time periods (1997 to 2003, 2004 to 2009, 2010 to 2012, and 2013 to 2015) reflecting major guideline changes.

**Results:**

From January 1997 to December 2015, there were 692 maternal deaths in the obstetrics unit. Of the 490 (70.8%) maternal deaths with a documented HIV status, 335 (68.4%) were HIV‐infected. A Chi‐squared test for trends showed that the institutional MMR (iMMR) in women known to be HIV‐infected peaked in the period 2004 to 2009 at 380 (95% CI 319 to 446) per 100,000 live births, with a decline to 267 (95% CI 198 to 353) in 2013 to 2015, *p *=* *0.049. This decrease coincided with changes in the South African HIV management guidelines, mainly increased availability of antiretroviral therapy (ART). Non‐pregnancy related infections were the leading cause of death throughout the review period, accounting for 61.5% (206/335) of deaths. Only 23.3% (78/335) of the women who died were on ART at the time of death, this in the context of advanced immune suppression and an overall median CD4 count of 136 cells/μl (interquartile ranges (IQR) 45 to 301).

**Conclusion:**

In this 19‐year review of maternal deaths in Johannesburg, South Africa, there was evidence of a decrease in the iMMR among HIV‐infected women, but it remains unacceptably high. Efforts to address drivers of mortality and barriers to accessing ART need to be accelerated if we are to see substantial decreases in maternal mortality.

## 1 Introduction

Decreasing maternal deaths remains a global priority. While progress has been made, many countries failed to achieve the Millennium Development Goal 5 (MDG 5) target of reducing the maternal mortality ratio (MMR) by 75%, between 1990 and 2015 1, 2. As work begins towards the Sustainable Development Goal target of reducing the global MMR to less than 70 deaths per 100,000 live births by 2030, much needs to be done in ending preventable maternal deaths 1, 3. While a large proportion of maternal deaths are still due to direct obstetric causes, such as haemorrhage, hypertension, sepsis, and complications of unsafe abortion, the past 20 years saw an increase in HIV‐related maternal deaths, with an estimated 90% of these occurring in sub‐Saharan Africa 1, 4.

South Africa, one of the sub‐Saharan countries with a high HIV prevalence among women of reproductive age, experienced an increase in the MMR in the late 1990s and in the first decade of the 2000s, largely due to the HIV epidemic 1, 5, 6. There has been some reduction in HIV‐related maternal deaths in the country since the scale‐up of antiretroviral therapy (ART) 6, 7. However, HIV infection still contributes significantly to maternal deaths in South Africa, with almost a third of deaths in 2015 estimated to be AIDS‐related 8.

HIV‐infected women experience excess pregnancy‐related mortality compared with HIV‐uninfected women 9, 10. A systematic review found an eight‐fold increased risk of mortality in HIV‐infected pregnant and postpartum women compared to uninfected women 9. In the review, only two studies from sub‐Saharan Africa included women on ART and most were initiated at very low CD4 counts 9. Hence, data are limited on the effect of ART on HIV‐attributable maternal mortality, in high HIV prevalence settings, as they predate the expansion of HIV programmes, evolution of HIV management guidelines and widespread availability of ART 9, 10. There remains a need to identify the leading causes of maternal mortality in HIV‐infected women, and also the characteristics of women who die, if we are to decrease HIV‐related maternal deaths 11.

In this 19‐year review, we assessed trends in maternal mortality and leading causes of death in HIV‐infected women, in a referral hospital in Johannesburg, South Africa, on a background of an evolving HIV care programme.

## 2 Methods

### 2.1 Study setting

The study was conducted at Chris Hani Baragwanath Academic Hospital (CHBAH), a referral hospital in Soweto, Johannesburg, an area of mixed urban and informal settlements. The obstetrics unit at CHBAH is a high‐volume unit, with 19,804 live births recorded for 2015. Low‐risk births are conducted in midwife‐run clinics in Soweto. Antenatal care is provided at around 60 community health centres, with referral of high‐risk pregnancies to the CHBAH antenatal clinic. Between 3% and 5% of women who give birth at CHBAH access antenatal care at the hospital (District Health Information System data). The area served by the hospital is a high HIV prevalence setting, and the HIV prevalence among pregnant women peaked at 30.7% in 2004, and has plateaued around 29.0% for the past decade (Unpublished PMTCT programme data). The figures are similar to those in published South African antenatal surveys 12.

South Africa has seen a rapid evolution of prevention of mother‐to‐child transmission of HIV (PMTCT) and ART guidelines. ARVs for PMTCT became available only in 2002, and between then and 2008, when short‐course antepartum zidovudine was introduced, only single‐dose nevirapine was available 13, 14. ART was introduced in South Africa in 2004 15, and universal coverage for ART in pregnant women, i.e. ≥80% coverage, was achieved in the Soweto PMTCT programme in 2012 (Unpublished PMTCT programme data). Up to 2013, when ART became available for all HIV‐infected pregnant women, CD4 count threshold or World Health Organization (WHO) clinical stage determined ART eligibility 16, 17. Option B+, lifelong triple therapy for all pregnant women, regardless of CD4 count or WHO stage, was adopted in South Africa in January 2015 18. HIV‐infected pregnant women seen at the Soweto facilities and at CHBAH were managed and continue to be managed according to the South African PMTCT and ART guidelines.

### 2.2 Study design

This was a cross‐sectional retrospective record review of maternal deaths in the obstetrics unit at CHBAH from January 1997 to December 2015. Since 1997, maternal deaths have been notifiable in South Africa, and the antenatal and hospital admission records for all maternal deaths have been kept safely in the offices of the obstetrics unit, at CHBAH. Data were extracted from these records and transcribed onto a structured data extraction tool. Data on the following parameters were collected: demographics, history of HIV diagnosis and management, details of antenatal care, admission details, laboratory and radiological investigations, delivery details, and clinical information about the circumstances of the deaths. The hospital registers and the National Health Laboratory Services (NHLS) were accessed for any information and laboratory test results that were missing in the patient records. Two obstetricians were involved in the data collection, verification and validation. One obstetrician viewed the original case notes and entered the data onto the extraction tool. The South African National Committee for Confidential Enquiries into Maternal Deaths (NCCEMD) classification was used to categorise causes of maternal deaths, and the two obstetricians were responsible for assigning the cause of death for each case 8. The NCCEMD provides ten disease categories for the cause of death and these are: hypertension, obstetric haemorrhage, ectopic pregnancy, miscarriage, pregnancy‐related sepsis, anaesthetic complications, embolism, acute collapse (cause unknown), non‐pregnancy related infections, and medical and surgical disorders. There is also a category for unknown, where the cause of death could not be determined. For each category, there are conditions listed to guide in determining the cause of death. The second obstetrician, who is a former member of the NCCEMD, reviewed all the data on the extraction tool and entered the data into Epi‐Info software^©^. Queries and disagreements about the causes of death were resolved by review of the case notes and consensus between the two obstetricians.

Results on the overall causes of maternal deaths and the MMR up to 2012 have been published elsewhere 7. This study analysed trends in maternal mortality and leading causes of deaths in HIV‐infected women, over different time periods reflecting evolving HIV management and PMTCT guidelines. The four time periods are: (1) 1997 to 2003: no ART was available for lifelong treatment, and only single‐dose nevirapine was available for PMTCT 13. All pregnant women were offered routine HIV testing and this period saw a rapid increase in the HIV prevalence among pregnant women attending antenatal care facilities in South Africa 12. (2) 2004 to 2009: HIV management guidelines in South Africa changed to introduce lifelong ART in 2004, with eligibility based on CD4 count ≤200 cells/μl or WHO stage 3 or 4 disease 15. Zidovudine monotherapy for PMTCT, to augment single‐dose nevirapine, was introduced in 2008 for women not eligible for ART 14. (3) 2010 to 2012: Routine third trimester retesting of pregnant women who initially test HIV negative was introduced 16. The CD4 count threshold for ART initiation in pregnant women increased to 350 cells/μl in 2010 16. This change in guideline, together with the introduction of nurse‐managed ART in 2012, increased the proportion of eligible pregnant women initiated on ART (Unpublished PMTCT programme data). (4) 2013 to 2015: all HIV‐infected pregnant and breastfeeding women were initiated on an efavirenz‐based fixed dose ART combination from 2013 and this was stopped postpartum if the woman did not fulfil criteria for lifelong treatment 17. From 2015, all HIV‐infected pregnant and breastfeeding women became eligible for lifelong ART 18. The retesting guidelines for women who initially test HIV negative were also updated in 2015 with introduction of routine retesting every three months during pregnancy and breastfeeding, with additional retesting intrapartum and six weeks postpartum 18. The study was approved by the Johannesburg's University of the Witwatersrand Human Research Ethics Committee, and by the hospital's medical advisory committee.

### 2.3 Data analysis

Data were categorised into the four time periods described above, and analyses were done with Stata^®^ version 13.0 (Stata Corporation, College Station, USA). Continuous data (gestational age, CD4 count, days in hospital) were summarised using medians with interquartile ranges (IQRs). All other variables were categorical and analysed using proportions and percentages, with 95% confidence intervals (CI) where appropriate. The overall institutional MMR (iMMR) was calculated using the number of live births at CHBAH, during the four time periods. To calculate the HIV status‐specific iMMR, the number of live births in HIV‐infected and HIV‐uninfected women was estimated using the HIV prevalence among pregnant women attending antenatal clinics in Soweto [12, unpublished PMTCT data]. The HIV prevalence was calculated for each of the four time periods. For the groups of women with unknown HIV status at the time of death, the iMMR could not be determined as no data were available on the proportion of women with unknown HIV status at the time of delivery. Therefore, a sensitivity analysis was done by imputing time‐period and cause‐of‐death specific HIV seropositivity proportions to women with unknown HIV status. Inferential analyses included tests of association by Chi‐squared or Fisher's exact tests for categorical data, and Chi‐squared test for trend where appropriate. The Wilcoxon rank‐sum test was used for comparison of continuous data. Statistical significance was accepted as the two‐tailed *p *<* *0.05.

## 3 Results

From January 1997 to December 2015, a total of 692 women died at CHBAH during pregnancy or within 42 days of delivery, of whom 490 (70.8%) had documented HIV status – 335 HIV‐infected and 155 HIV‐uninfected. The demographics of all the maternal deaths during the study period are presented in Table 1. Just over a half of all deaths, 55.0% (376/684), occurred in the age group 25 to 35 years, with a higher proportion of deaths among HIV‐infected women in this age group at 60.9% (204/335). In the 614 maternal deaths with antepartum information, 76.4% (n = 469) accessed antenatal care, with the majority accessing care at a gestational age of 20 weeks or later. There was an increase in the proportion of HIV‐infected maternal deaths who had no antenatal care in the later time periods, with almost one‐third from 2013 to 2015 not accessing antenatal care (*p *=* *0.008). Also, a high proportion of women with unknown HIV status had not accessed antenatal care throughout the review period.

**Table 1:** Demographic data in maternal deaths of HIV‐infected and HIV‐uninfected women, and women with unknown HIV status (n; %), n = 692

	1997 to 2003			2004 to 2009			2010 to 2012			2013 to 2015		
	HIV‐infected (n = 71)	HIV‐uninfected (n = 30)	HIV unknown (n = 107)	HIV‐infected (n = 144)	HIV‐uninfected (n = 63)	HIV unknown (n = 61)	HIV‐infected (n = 71)	HIV‐uninfected (n = 25)	HIV unknown (n = 18)	HIV‐infected (n = 49)	HIV‐uninfected (n = 37)	HIV unknown (n = 16)
Age (n = 684)												
<18	2 (2.8)	1 (3.3)	4 (4.0)	2 (1.4)	2 (3.2)	1 (1.7)	2 (2.8)	2 (8.0)	1 (5.6)	0 (0.0)	0 (0.0)	1 (6.2)
18 to 24	25 (35.2)	1 (3.3)	23 (23.0)	21 (14.6)	20 (31.8)	13 (21.7)	11 (15.5)	5 (20.0)	2 (11.1)	7 (14.3)	13 (35.1)	3 (18.8)
25 to 35	36 (50.7)	18 (60.0)	49 (49.0)	94 (65.3)	28 (44.4)	30 (50.0)	43 (60.6)	12 (48.0)	11 (61.1)	31 (63.3)	18 (48.6)	6 (37.5)
>35	8 (11.3)	10 (33.3)	24 (24.0)	27 (18.7)	13 (20.6)	16 (26.6)	15 (21.1)	6 (24.0)	4 (22.2)	11 (24.4)	6 (16.2)	6 (37.5)
Parity (n = 648)												
0	23 (23.3)	5 (17.9)	24 (25.3)	33 (23.6)	28 (45.2)	9 (16.7)	12 (17.4)	7 (28.0)	4 (33.3)	6 (13.0)	14 (38.9)	3 (25.0)
1 to 4	45 (65.2)	18 (64.3)	64 (67.3)	103 (73.7)	31 (50.0)	42 (77.8)	56 (81.2)	17 (68.0)	8 (66.7)	39 (84.8)	20 (55.6)	9 (75.0)
≥5	1 (1.5)	5 (17.8)	7 (7.4)	4 (2.8)	3 (4.8)	3 (5.5)	1 (1.4)	1 (4.0)	0 (0.0)	1 (2.2)	2 (5.5)	0 (0.0)
Accessed antenatal care (n = 614)												
Yes	61 (85.9)	19 (63.3)	58 (54.2)	106 (73.6)	57 (90.5)	24 (39.3)	50 (70.4)	23 (92.0)	3 (16.7)	33 (67.3)	35 (94.6)	0 (0.0)
No	6 (8.5)	2 (6.7)	33 (30.8)	26 (18.1)	5 (7.9)	23 (37.7)	13 (18.3)	0 (0.0)	12 (66.6)	14 (28.6)	1 (2.7)	10 (62.5)
Unknown	4 (5.6)	9 (30.0)	16 (15.0)	12 (8.3)	1 (1.6)	14 (23.0)	8 (11.3)	2 (8.0)	3 (16.7)	2 (4.1)	1 (2.7)	6 (37.5)
Gestational age at accessing antenatal care (n = 428)												
<20 weeks	27 (47.4)	5 (29.4)	14 (26.4)	34 (35.1)	19 (38.0)	5 (21.7)	12 (26.7)	10 (43.5)	2 (66.7)	15 (53.6)	19 (59.4)	0 (0.0)
≥20 weeks	30 (52.6)	12 (70.6)	39 (73.6)	63 (64.9)	31 (62.0)	18 (78.3)	33 (73.3)	13 (56.5)	1 (33.3)	13 (46.4)	13 (40.6)	0 (0.0)

While 83.8% of women (281/335) were diagnosed as HIV‐infected during pregnancy, trends in HIV diagnosis changed over time. The HIV testing rate increased from 48.5% (101/208) in 1997 to 2003, to 84.3% (86/102) in 2013 to 2015 (*p *<* *0.001). Pre‐pregnancy diagnosis of HIV infection increased from 2.8% (2/71) in 1997 to 2003, to 34.7% (17/49) in 2013 to 2015 (*p *<* *0.001; Table 2). Assessment for ART eligibility with an antepartum CD4 count also increased from 8.4% (6/71) in 1997 to 2003, with a peak in testing of 70.4% (50/71) in 2010 to 2012. There was a decline in CD4 testing in 2013 to 2015, coinciding with guideline changes where all HIV‐infected pregnant women became eligible for ART. Overall, 133 women (39.7%) had an antepartum CD4 count, with a median of 136 cells/μl (IQR 45 to 301 cells/μl). There was a non‐significant trend towards an increase in the median antepartum CD4 count from 102 cells/μl (IQR 56 to 289) in 1997 to 2003 to 220 cells/μl (IQR 61 to 361) in 2013 to 2015 (*p *=* *0.071), and also a trend towards a decrease in the proportion of women with advanced immune suppression (Table 2). While ART initiation peaked in the period 2010 to 2012, there was a decline in the proportion of women initiated on ART in 2013 to 2015, when all HIV‐infected women were eligible for ART. In the 19 women who were not started on ART in the period 2013 to 2015, the median antepartum CD4 count was 173 cells/μl (IQR 16 to 340), and 12 of the 19 women did not access antenatal care.

**Table 2:** Diagnosis and management of HIV infection, n = 335

	1997 to 2003 (n = 71)	2004 to 2009 (n = 144)	2010 to 2012 (n = 71)	2013 to 2015 (n = 49)	*p*‐value
Timing of HIV diagnosis, n (%)					
Pre‐pregnancy	2 (2.8)	4 (2.8)	4 (5.6)	17 (34.7)	<0.001
Antepartum	61 (86.0)	129 (89.6)	66 (93.0)	25 (51.0)	
Intrapartum/postpartum	7 (9.8)	9 (6.2)	0 (0.0)	4 (8.2)	
Unknown	1 (1.4)	2 (1.4)	1 (1.4)	3 (6.1)	
Antenatal CD4 count done, n (%)					
Yes	6 (8.4)	52 (36.1)	50 (70.4)	25 (51.0)	<0.001
No	65 (91.6)	92 (63.9)	21 (29.6)	24 (49.0)	
Nadir CD4 count, cells/μl, n (%)^a^					
Total done	24 (33.8)	102 (70.8)	64 (90.1)	32 (65.3)	<0.001
<100	17 (70.8)	62 (60.8)	28 (43.8))	14 (43.7)	0.061
100 to 200	3 (12.5)	19 (18.6)	13 (20.3)	6 (18.7)	
201 to 350	1 (4.2)	12 (11.8)	18 (28.1)	6 (18.7)	
>350	3 (12.5)	9 (8.8)	5 (7.8)	6 (18.7)	
ART eligible, n (%)^b^	0 (0.0)	37/52 (71.1)	41/50 (82.0)	49/49 (100.0)	<0.001
Initiated on ART, n (%)	0 (0.0)	19 (51.4)	29 (70.7)	30 (61.2)	0.600
Timing of ART, n (%)					
Preconception	0 (0.0)	6 (4.2)	7 (9.8)	14 (28.6)	<0.001
Antepartum	0 (0.0)	13 (9.0)	22 (31.0)	16 (32.7)	
Not started/ unknown	71 (100.0)	125 (86.8)	42 (59.2)	19 (38.7)	

a Nadir CD4 count done either antenatally or during admission when the death occurred.

b ART, antiretroviral therapy eligibility: in 1997 to 2003, no women; in 2004 to 2009, women with CD count ≤200 cells/μl; in 2010 to 2012, women with CD4 count ≤350 cells/μl; in 2013 to 2015, all HIV‐infected women irrespective of CD4 count.

Overall, 75.3% of women (521/692) were still pregnant at the time of admission, while 75.7% of deaths (524/692) occurred postpartum. The proportions were similar for HIV‐infected women, with 75.2% (252/335) admitted either antenatally or intrapartum and 79.1% (265/335) dying postpartum – Table 3. Women with unknown HIV status were more likely to die within the first 24 hours of admission, with a median time from admission to death of one day (IQR 0 to 3), less than in women with known HIV status, who died after a median of three days (IQR 1 to 7), *p *<* *0.001. Among HIV‐infected women, the median time from admission to death was three days (IQR 1 to 7), while that in HIV‐uninfected women was two days (IQR 1 to 6), *p *=* *0.011. The median gestational age at delivery or death was lower among HIV‐infected women (31 weeks; IQR 27 to 35 weeks) compared with HIV‐uninfected women (35 weeks; IQR 31 to 38 weeks), *p *<* *0.001. In the later time periods, women with unknown HIV status died at a relatively early gestational age, median of 28 weeks (IQR 18 to 35) in 2010 to 2012, and 17 weeks (IQR 10 to 26) in 2013 to 2015 (Table 3).

**Table 3:** Timing of admission and timing of death (n;%), n = 692

	1997 to 2003			2004 to 2009			2010 to 2012			2013 to 2015		
	HIV‐infected (n = 71)	HIV‐uninfected (n = 30)	HIV unknown (n = 107)	HIV‐infected (n = 144)	HIV‐uninfected (n = 63)	HIV unknown (n = 61)	HIV‐infected (n = 71)	HIV‐uninfected (n = 25)	HIV unknown (n = 18)	HIV‐infected (n = 49)	HIV‐uninfected (n = 37)	HIV unknown (n = 16)
Timing of admission												
Antenatal	44 (62.0)	16 (53.3)	71 (66.3)	75 (52.1)	33 (52.4)	24 (39.3)	46 (64.8)	14 (56.0)	10 (55.5)	27 (55.1)	23 (62.2)	12 (75.0)
Intrapartum	14 (19.7)	5 (16.7)	16 (15.0)	32 (22.2)	15 (23.8)	18 (29.5)	6 (8.5)	5 (20.0)	3 (16.7)	8 (16.3)	4 (10.8)	0 (0.0)
Postpartum	13 (18.3)	9 (30.0)	20 (18.7)	37 (25.7)	15 (23.8)	19 (31.1)	19 (26.7)	6 (24.0)	5 (27.8)	14 (28.6)	10 (27.0)	4 (25.0)
Timing of death												
Antenatal	13 (18.3)	6 (20.0)	28 (26.2)	29 (20.1)	6 (9.5)	14 (23.0)	13 (18.3)	3 (12.0)	8 (44.4)	11 (22.4)	5 (13.5)	3 (18.8)
Postpartum	58 (81.7)	24 (80.0)	79 (73.8)	115 (79.9)	57 (90.5)	47 (77.0)	58 (81.7)	22 (88.0)	10 (55.6)	38 (77.6)	32 (86.5)	13 (81.2)
Days from admission to death, n (%)												
0 to 1	15 (21.1)	10 (33.3)	56 (52.3)	45 (31.2)	33 (52.4)	41 (67.2)	18 (25.4)	12 (48.0)	11 (61.1)	17 (34.7)	11 (29.7)	7 (43.7)
2 to 4	27 (38.0)	9 (30.0)	23 (21.5)	38 (26.4)	17 (27.0)	12 (19.7)	27 (38.0)	5 (20.0)	5 (27.8)	13 (26.5)	11 (29.7)	6 (37.5)
5 to 9	16 (22.5)	5 (16.7)	14 (13.1)	37 (25.7)	8 (12.7)	5 (8.2)	17 (23.9))	5 (20.0)	1 (5.5)	11 (22.4)	10 (27.0)	3 (18.8)
>9	13 (18.3)	6 (20)	14 (13.1)	24 (16.7)	5 (7.9)	3 (4.9)	9 (12.7)	3 (12.0)	1 (5.5)	8 (16.3)	5 (13.5)	0 (0.0)
Gestational age at delivery or death (weeks), n = 602 (%)												
Median (IQR)	31 (28 to 34)	34 (28 to 37)	32 (26 to 36)	31 (26 to 36)	35 (30 to 39)	32 (25 to 38)	32 (28 to 36)	34 (32 to 38)	28 (18 to 35)	31 (25 to ‐35)	36 (32 to 39)	17 (10 to 26)
<22	4 (6.0)	2 (8.0)	12 (13.6)	4 (3.2)	1 (1.7)	5 (10.8)	6 (9.4)	0 (0.0)	4 (30.8)	6 (14.3)	3 (8.1)	7 (58.3)
22 to 27	11 (16.4)	2 (8.0)	14 (16.0)	33 (26.2)	5 (8.5)	9 (19.6)	10 (15.6)	3 (13.0)	2 (15.4)	7 (16.7)	2 (5.4)	3 (25.0)
28 to 36	43 (64.2)	14 (56.0)	41 (46.6)	60 (47.6)	30 (50.8)	16 (34.8)	34 (53.1)	11 (47.8)	5 (38.4)	19 (45.2)	15 (40.5)	2 (16.7)
>36	9 (13.4)	7 (28.0)	21 (23.9)	29 (23.0)	23 (39.0)	16 (34.8)	14 (21.9)	9 (39.1)	2 (15.4)	10 (23.8)	17 (45.9)	0 (0.0)

IQR, interquartile ranges.

The causes of death in all women who died during the study period are presented in Table 4. Non‐pregnancy related infections were the leading cause of death in HIV‐infected women throughout the review period, accounting for 61.5% (206/335) of deaths. The proportion of deaths due to these infections, among HIV‐infected women, peaked in 1997 to 2003 at 69.0% (49/71), with a decrease to 44.9% (22/49) in 2013 to 2015, *p *<* *0.001 (Table 2). Among the non‐pregnancy related infections, the leading causes of death were respiratory infections, accounting for 56.3% (116/206) of the deaths. Tuberculosis, pulmonary and extrapulmonary, accounted for 32.5% (67/206) of the non‐pregnancy related infections. Of the HIV‐infected women who died of non‐pregnancy related infections and had antepartum CD4 count testing, 79.3% (65/82) had a CD4 count ≤200 cells/μl, compared with 31.4% (16/51) among women who died from other causes (*p *<* *0.001). A total of 46/206 (22.3%) HIV‐infected women who died of non‐pregnancy related infections were on ART at the time of death. Among the HIV‐infected women who died of pregnancy‐related sepsis, the median antepartum CD4 count was 236 cells/μl (IQR 110 to 325), and 10/25 (40.0%) were on ART. Puerperal sepsis following caesarean section accounted for 64.0% (16/25) of cases of pregnancy‐related sepsis among HIV‐infected women. Complications related to hypertensive disorders were the leading cause of death among HIV‐uninfected women and women with unknown HIV status. Autopsies were performed in 2.6% (18/692) of the maternal deaths.

**Table 4:** Causes of maternal death in HIV‐infected and HIV‐uninfected women, and women with unknown HIV status (n; %), n = 692

	1997 to 2003			2004 to 2009			2010 to 2012			2013 to 2015		
	HIV‐infected (n = 71)	HIV‐uninfected (n = 30)	HIV unknown (n = 107)	HIV‐infected (n = 144)	HIV‐uninfected (n = 63)	HIV unknown (n = 61)	HIV‐infected (n = 71)	HIV‐uninfected (n = 25)	HIV unknown (n = 18)	HIV‐infected (n = 49)	HIV‐uninfected (n = 37)	HIV unknown (n = 16)
Non‐pregnancy related infections	49 (69.0)	8 (26.7)	11 (10.3)	93 (64.6)	8 (12.7)	9 (14.7)	42 (59.1)	2 (8.0)	2 (11.1)	22 (44.9)	5 (13.5)	0 (0.0)
Obstetric haemorrhage	3 (4.2)	3 (10.0)	20 (18.7)	16 (11.1)	11 (17.5)	13 (21.3)	7 (9.8)	4 (16.0)	2 (11.1)	4 (8.2)	6 (16.2)	1 (6.3)
Pregnancy‐related sepsis	3 (4.2)	6 (20.0)	4 (3.7)	11 (7.6)	6 (9.5)	0 (0.0)	5 (7.0)	3 (12.0)	0 (0.0)	6 (12.2)	5 (13.5)	0 (0.0)
Hypertensive disorders	2 (2.8)	7 (23.3)	31 (29.0)	9 (6.3)	18 (28.6)	21 (34.4)	2 (2.8)	5 (20.0)	8 (44.4)	9 (18.4)	9 (24.3)	3 (18.7)
Medical and surgical disorders	2 (2.8)	4 (13.3)	10 (9.3)	3 (2.1)	10 (15.9)	3 (4.9)	10 (14.1)	5 (20.0)	2 (11.1)	4 (8.2)	11 (29.7)	2 (12.5)
Other^a^	9 (12.7)	2 (6.7)	25 (23.4)	12 (8.3)	8 (12.7)	13 (21.3)	3 (4.2)	5 (20.0)	4 (22.2)	3 (6.1)	1 (2.7)	10 (62.5)
Unknown	3 (4.2)	0 (0.0)	6 (5.6)	0 (0.0)	2 (3.2)	2 (3.3)	2 (2.8)	1 (4.0)	0 (0.0)	1 (2.0)	0 (0.0)	0 (0.0)

a Other causes of death included: complications of miscarriages and ectopic pregnancies, anaesthetic deaths and cases of acute collapse that include suspected thromboembolism.

The majority of the autopsies were done in cases of acute collapse, and in cases of medical and surgical disorders complicating pregnancy, where the cause of death was not clear, or of clinical interest.

The iMMR in HIV‐infected women peaked in the period 2004 to 2009 at 380 per 100,000 live births, with a decline to 267 in 2013 to 2015, *p *=* *0.049 (Table 5). It was three‐ to eightfold higher in the different time periods compared to the iMMR in HIV‐uninfected women. The overall iMMR decreased from 209 per 100,000 live births in 2004 to 2009, to 162 in 2013 to 2015 (*p *=* *0.02). The iMMR showed an upward trend in HIV‐uninfected women (*p *<* *0.001). A sensitivity analysis was done to determine the effect of unknown HIV status on these findings. Observed proportions of HIV infections were imputed for the unknown groups in each cause‐of‐death category, in each time period. For example, in 1997 to 2003, the HIV‐infected proportion for women with non‐pregnancy related infections was 49/57 (0.86, Table 4). This proportion was applied to the 11 women whose HIV status was unknown, giving nine HIV‐infected and two HIV‐uninfected women. The process was repeated for all seven cause‐of‐death categories as shown, for each of the four time periods. For women thus assumed HIV‐infected, the iMMR decreased from 476 to 464 to 413 to 322 per 100,000 live births in the four time periods starting 1997 to 2003 (*p *=* *0.01). For women assumed HIV‐uninfected, the corresponding trend was 86 to 101 to 73 to 97 per 100,000 live births (*p *=* *0.91), suggesting a stable iMMR.

**Table 5:** Institutional maternal mortality ratios at Chris Hani Baragwanath Academic Hospital, 1997 to 2015

	1997 to 2003	2004 to 2009	2010 to 2012	2013 to 2015
HIV prevalence, %	22.0	29.6	28.5	29.2
Live births, n				
Total live births	121,353	128,150	67,056	62,785
Estimated live births in HIV‐infected women	26,698	37,932	19,111	18,333
Estimated live births in HIV‐uninfected women	94,655	90,218	47,945	44,452
iMMR (95% CI)				
Overall iMMR^a^	171 (149 to 196)	209 (185 to 236)	170 (140 to 204)	162 (132 to 197)
iMMR in HIV‐infected women	267 (208 to 335)	379 (319 to 446)	372 (290 to 468)	267 (198 to 353)
iMMR in HIV‐uninfected women	32 (21 to 45)	70 (54 to 89)	52 (34 to 77)	83 (59 to 115)
Cause‐specific iMMR (95% CI)^a^				
Non‐pregnancy related infections	56 (44 to 71)	86 (71 to 103)	69 (50 to 91)	43 (28 to 63)
All causes of death, excluding non‐pregnancy related infections and unknown cause	108 (90 to 128)	120 (101 to 140)	97 (75 to 124)	118 (93 to 148)

a Includes maternal deaths with unknown HIV status.

## 4 Discussion

In this 19‐year review of the evolution of HIV diagnosis and management in pregnancy, and trends in maternal deaths, the iMMR in HIV‐infected women peaked in 2004 to 2009, followed by a progressive decline in the last six years of the review period, with a more substantial decrease in 2013 to 2015. This decrease in the iMMR coincides with several changes in the South African PMTCT and HIV management guidelines 13, 14, 15, 16, 17, 18. The overall iMMR showed a similar trend to that in HIV‐infected women, while that in HIV‐uninfected women showed no downward trend. This highlights the importance of deaths in HIV‐infected women in influencing trends in maternal mortality, and is also reflected in the South African National Confidential Enquiries into maternal deaths, which also report on the iMMR 6. Data from the National Confidential Enquiries for the period 2011 to 2013 show a decline in the iMMR largely related to a decrease in deaths due to non‐pregnancy related infections in whom 90% of the women were HIV‐infected 6. In our study, the proportion of maternal deaths with known HIV status increased over time, with an increase in pre‐pregnancy diagnosis of HIV infection, but the majority were still diagnosed during pregnancy. While there was a significant increase in ART initiation, the majority of women died without ever starting ART, this in the context of advanced immune suppression. Non‐pregnancy related infections remained the leading cause of maternal deaths in HIV‐infected women throughout the review period. In the later periods, the characteristics of women with unknown HIV status had become more distinct from those who had tested. HIV unknown women more frequently did not access antenatal care, suffered early pregnancy complications, and died soon after admission to hospital – all reasons for not being tested for HIV.

The majority of women who died had accessed antenatal care, but a high proportion of women with an unknown HIV status had not accessed any care. There was also a trend towards a relative increase in the proportion of maternal deaths known to be HIV‐infected who had no antenatal care – almost a third in 2013 to 2015. The latest available figure for antenatal care coverage in South Africa is for 2014, and the reported coverage is 90% 19. Our finding on rates of antenatal care attendance among HIV‐infected women and women of unknown HIV status are hence lower than the national average, but similar to that reported in a review of maternal deaths in South Africa at the early stages of ART roll‐out 20. Black *et al*. 20, in their five‐year review of maternal deaths at a tertiary hospital, also found a high rate of nonattendance for antenatal care among the women who died. Our finding of an increase in HIV‐infected women not accessing antenatal care in 2013 to 2015 is a reflection of the women who remain at risk of dying. Failure to access antenatal care is of concern as antenatal care remains the cornerstone for detection of HIV infection and identification of obstetric complications. Although antenatal care cannot impact on all causes of maternal mortality, it can have a significant impact in decreasing HIV‐related mortality in high HIV prevalence settings, with early diagnosis and treatment of pregnant women identified as HIV‐infected 21, 22. In our study, the majority of HIV‐infected women were diagnosed during pregnancy and this points to a need to increase routine HIV testing outside of pregnancy to allow linkage to care and early initiation of ART. Preconception ART is associated with a decreased risk of HIV‐related maternal deaths 23, 24.

There was a fall‐off at all stages of the HIV management cascade, from HIV testing to assessment for ART eligibility, to initiation of treatment in those found to be eligible. In 2004 to 2012, with introduction and scale‐up of availability of ART, less than half of the women had a CD4 count done to assess for ART eligibility. This highlights deficiencies in provision of services and delays in linkage to care. The number of women who were on ART at the time of death increased over time, but less than a quarter were initiated on ART overall, despite advanced immune suppression where the overall median CD4 count was 136 cells/μl. Even in 2013 to 2015 when all HIV‐infected pregnant women were eligible for ART, 38.8% were not on treatment at the time of death. Missed opportunities along the HIV management cascade not only impact on maternal health, but have also been directly linked to the risk of MTCT 25. In a South African national survey, a third of early infant HIV infections were found to be attributable to missed opportunities along the cascade 25. Historically, delays in ART initiation during pregnancy were related to complex evaluation processes before starting treatment and the lack of integration of antenatal and ART services 26. While ART initiation has been simplified, and integration of services has increased the proportion of pregnant women started on treatment, there are still individual and contextual factors that impact on ART initiation and retention in care 26, 27, 28.

While a large proportion of HIV‐infected women who died presented in the antenatal period, the majority of deaths occurred postpartum, and in most cases the conditions that led to the deaths occurred in the antenatal and intrapartum periods. This finding was similar among HIV‐uninfected women and women with unknown HIV status. In our study, the leading cause of death among HIV‐uninfected women and women with unknown HIV status were complications related to hypertensive disorders. Among HIV‐infected women, almost two‐thirds of maternal deaths were due to non‐pregnancy related infections, the majority respiratory infections, including TB. This finding is consistent with data from reports on the Confidential Enquiries into Maternal Deaths in South Africa 6. There was however a progressive decline in maternal deaths due to non‐pregnancy related infections, starting in 2010 to 2012 and coinciding with the increase in CD4 count threshold for ART initiation in pregnant women and a greater proportion initiated on ART through integration of services. Respiratory infections are among the most important non‐pregnancy related infectious causes of death among HIV‐infected women 22, 29. Physiologic and immunologic changes in pregnancy, and immune suppression related to HIV infection, are all thought to increase susceptibility to infections, including respiratory infections 22. In our study, no data were available on the use of cotrimoxazole and isoniazid, both effective prophylactic interventions in HIV‐infected individuals 30. Data from South Africa indicate poor uptake of isoniazid preventative therapy among HIV‐infected pregnant women 31, 32. There have been concerns about the risk of hepatitis with isoniazid use in pregnancy, but available evidence suggests no increased risk even in pregnant women on ART receiving long‐term isoniazid 33.

Among the pregnancy‐related causes of maternal deaths, a threefold increased risk of puerperal sepsis has been reported in HIV‐infected women, and the risk is said to be even higher, almost six times, following a caesarean section, consistent with our findings where the majority of cases of puerperal sepsis were secondary to caesarean sections 34. The increased risk of puerperal sepsis has been attributed to the immune suppression associated with HIV infection 34. Although the median CD4 count among women who died of puerperal sepsis in our study was higher than the median CD4 count overall, less than half were on ART at the time of death. Due to the limited number of published studies, it is not possible to determine the effect of ART on the risk of puerperal sepsis 34. Data on the association between other obstetric complications and HIV infection are inconsistent 34, 35. There is hence a need for improvements in identification and management of obstetric complications that may be aggravated by underlying HIV infection 11. There is also a need to address the unmet need for contraception in both HIV‐infected and uninfected women as prevention of unintended pregnancies also contributes to the reduction in maternal deaths 2.

It is widely recognised that early initiation of ART needs to be scaled up if we are to see a sustained reversal in HIV‐related maternal deaths. At the end of our review period, it was still too soon to see the impact of lifelong ART for all pregnant and postpartum women. As ART programmes are scaled up, there is a need for strategies to increase retention in care, especially postpartum – a period associated with high rates of loss‐to‐follow‐up 28. Integration of maternal health and ART services, facility‐ and community‐based support programmes, and male partner involvement are all strategies that have been used in improving retention in care 36. In our study, there was limited information on women who had defaulted ART. Data from Malawi, one of the first countries to introduce Option B+, indicate high rates of loss to follow‐up in the first year of starting ART, especially with ART initiation during pregnancy and when there was no maternal indication to start treatment 37.

There are several strengths to our study, and these include a long review period with data spanning almost two decades, starting in the pre‐ART era until ART became standard of treatment for all HIV‐infected pregnant women. The other strength is the vigilance in storage of records of women who died in our obstetrics unit. A standardised classification for causes of death was also used, minimising the chances of misclassifications of maternal deaths. The main limitation was the absence of HIV status in a large proportion of women who died in the early years of the study period, giving artefactually low estimates of iMMR. In our study, 68% of women who died with a known HIV status were HIV‐infected. Hence, the HIV prevalence in women with unknown HIV status could have been anywhere between 29%, the prevalence rate in pregnant women attending antenatal clinics, and 68%, the prevalence rate in women who died. The sensitivity analysis, with its given assumption, gave higher estimates of iMMR, especially for 1997 to 2003, but the significant downward trend for HIV‐infected women remained, and appears to be a robust finding. Another limitation is the absence of information on deaths occurring outside the hospital obstetrics unit, but we consider this proportion to be small 7. Ideally, this study should have included comparative data on the many thousands of HIV‐infected women who gave birth at the hospital and survived. Gathering such information was beyond our capacity and mostly impossible, because hospital records were destroyed after five years of storage during most of the study periods.

## 5 Conclusions

With scaling up of ART availability and better integration of antenatal and HIV management services in South Africa, we are starting to see a positive impact on maternal mortality in HIV‐infected women. However, the MMR in HIV‐infected women remains unacceptably high. Efforts to address drivers of mortality in this group of women, and barriers to accessing ART that still exist, need to be accelerated if we are to see substantial decreases in maternal mortality.

## Competing interests

The authors report no conflicts of interest.

## Authors’ contributors

CNM initiated the study, contributed to the study design and data collection, and was the main investigator responsible for the interpretation of results and drafting of the manuscript. EJB contributed to the study design, data collection and analysis, and drafting of the manuscript. MFC contributed to the data analysis and drafting of the manuscript. KF contributed to the study design and led the data collection. JAM contributed to the study design and drafting of the manuscript. All authors reviewed, contributed to and approved the final manuscript.

## Funding

The study was funded by the Carnegie Corporation of New York PhD Fellowship (grant number: B 8749.RO1), SACEMA (DST/NRF Centre of Excellence in Epidemiological Modelling and Analysis), Stellenbosch University, and Anova Health Institute through the US President's Emergency Plan for AIDS Relief (PEPFAR) via the US Agency for International Development, under Cooperative Agreement No. 674‐A‐00‐08‐00,009‐00. The funders had no role in interpretation of data, or writing of the manuscript, and the views expressed in the manuscript are those of the authors.

## References

[1] Alkema L , Chou D , Hogan D , Zhang S , Moller A , Gemmill A ; , et al. Global, regional, and national levels and trends in maternal mortality between 1990 and 2015, with scenario‐based projections to 2030: a systematic analysis by the UN Maternal Mortality Estimation Inter‐Agency Group. Lancet. 2016; 387:462–74. 26584737 10.1016/S0140-6736(15)00838-7 PMC5515236

[2] United Nations . The Millennium Development Goals Report 2015. New York, 2015 [accessed 2016 June 22]. Available from: http://www.un.org/millenniumgoals/2015_MDG_Report/pdf/MDG%202015%20rev%20(July%201).pdf

[3] Chou D , Daelmans B , Jovilet RR , Kinney M , Say L ; Every Newborn Action Plan (ENAP) and Ending Preventable Maternal Mortality (EPMM) working groups . Ending preventable maternal and newborn mortality and stillbirths. BMJ. 2015; 351:h4255. 26371222 10.1136/bmj.h4255

[4] Say L , Chou D , Gemmill A , Tunçalp Ö , Moller A , Daniels J , et al. Global causes of maternal deaths: a WHO systematic analysis. Lancet Glob Health. 2014;2:e323–33. 25103301 10.1016/S2214-109X(14)70227-X

[5] Hogan MC , Foreman KJ , Naghavi M , Ahn SY , Wang M , Makela AM , et al. Maternal mortality for 181 countries, 1980‐2008: a systematic analysis of progress towards Millennium Development Goal 5. Lancet. 2010;375:1609–23. 20382417 10.1016/S0140-6736(10)60518-1

[6] National Committee for the Confidential Enquiries into Maternal Deaths . Tenth interim report on confidential enquiries into maternal deaths in South Africa, 2011 and 2012. Pretoria: National Department of Health; 2013 [accessed 2016 May 18]. Available from: http://www.rmchsa.org/wp-content/uploads/2013/05/Tenth-Interim-Report-Maternal-Deaths-2011-and-2012.pdf

[7] Buchmann EJ , Mnyani CN , Frank KA , Chersich MF , McIntyre JA . Declining maternal mortality in the face of persistently high HIV prevalence in a middle‐income country. BJOG. 2014;122(2):220–7. 25213804 10.1111/1471-0528.13064

[8] World Health Organization . Trends in maternal mortality: 1990 to 2015: estimates by WHO, UNICEF, UNFPA, World Bank Group and the United Nations Population Division. Geneva: World Health Organization; 2015 [accessed 2016 June 22] http://apps.who.int/iris/bitstream/10665/194254/1/9789241565141_eng.pdf?ua=1

[9] Calvert C , Ronsmans C . The contribution of HIV to pregnancy‐related mortality: a systematic review and meta‐analysis. AIDS. 2013;27:1631–9. 23435296 10.1097/QAD.0b013e32835fd940 PMC3678884

[10] Zaba B , Calvert C , Marston M , Isingo R , Nakiyingi‐Miiro J , Lutalo J , et al. Effect of HIV infection on pregnancy‐related mortality in sub‐Saharan Africa: secondary analyses of pooled community‐based data from the network for Analysing Longitudinal Population‐based HIV/AIDS data on Africa (ALPHA). Lancet. 2013;381:1763–71. 23683643 10.1016/S0140-6736(13)60803-X PMC4325135

[11] Kendall T , Danel I , Cooper D , Dilmitis S , Kaida A , Kourtis AP , et al. Eliminating preventable HIV‐related maternal mortality in Sub‐Saharan Africa: what do we need to know? J Acquir Immune Defic Syndr. 2014;67:S250–8. 25436825 10.1097/QAI.0000000000000377 PMC4251907

[12] The 2012 National Antenatal Sentinel HIV and Herpes Simplex type‐2 prevalence Survey. South Africa: National Department of Health 2013 [accessed 2016 May 15]. Available from: https://www.health-e.org.za/wp-content/uploads/2014/05/ASHIVHerp_Report2014_22May2014.pdf

[13] Protocol for providing a comprehensive package of care for the prevention of mother‐to‐child transmission of HIV (PMTCT) in South Africa. Pretoria: Department of Health; 2001 [accessed 2016 May 15]. Available from: www.healthlink.org.za/uploads/files/nationalpro01.doc

[14] National Department of Health . Policy and Guidelines for the Implementation of the PMTCT Programme. Pretoria: South African National Department of Health; 2008. [accessed 2016 March 22].

[15] National antiretroviral treatment guidelines. Pretoria: Department of Health ; 2004 [accessed 2016 March 22]. Available from: http://www.doh.gov.za/docs/facts/2004/intro.pdf

[16] National Department of Health, South Africa; South African National AIDS Council . Clinical guidelines: PMTCT (Prevention of Mother‐to‐Child Transmission), 2010. Pretoria: South African National Department of Health; 2010 [accessed 2016 March 22 ). Available from: http://www.sahivsoc.org/upload/documents/NDOH_PMTCT.pdf

[17] Revised Antiretroviral Treatment Guideline Update for Frontline Clinical Health Professionals. South African National Department of Health; 2013 [accessed 2016 March 24 ]. Available from: http://www.sahivsoc.org/upload/documents/FDC%20Training%20Manual%2014%20March%202013(1).pdf

[18] National Consolidated Guidelines for the prevention of mother‐to‐child transmission of HIV (PMTCT) and the management of HIV in children, adolescents and adults. South African National Department of Health; 2015 [accessed 2016 24 March]. Available from: http://www.sahivsoc.org/upload/documents/HIV%20guidelines%20_Jan%202015.pdf

[19] Day C , Gray A . Health and related indicators In: Padarath A, King J, Mackie E, Casciola J, editors. South African Health Review 2016. Durban: Health Systems Trust; 2016 Available from: http://www.hst.org.za/publications/south-african-health-review-2016

[20] Black V , Brooke S , Chersich MF . Effect of human immunodeficiency virus treatment on maternal mortality at a tertiary center in South Africa: a 5‐year audit. Obstet Gynecol. 2009;114(2):292–9. 19622990 10.1097/AOG.0b013e3181af33e6

[21] Oyerinde K . Can antenatal care result in significant maternal mortality reduction in developing countries? J Community Med Health Educ. 2013;3:e116.

[22] Lathrop E , Jamieson DJ , Danel I . HIV and maternal mortality. Int J Gynaecol Obstet. 2014;127(2):213–5. 25097142 10.1016/j.ijgo.2014.05.024 PMC4302331

[23] Liotta G , Mancinelli S , Nielsen‐Saines K , Gennaro E , Scarcella P , Magid NA , et al. Reduction of maternal mortality with highly active antiretroviral therapy in a large cohort of HIV‐infected pregnant women in Malawi and Mozambique. PLoS One. 2013;8(8):e71653. 23990966 10.1371/journal.pone.0071653 PMC3747183

[24] Holtz SA , Thetard R , Konopka SN , Albertini J , Amzel A , Fogg KP . A systematic review of interventions to reduce maternal mortality among HIV‐infected pregnant and postpartum women. Int J of MCH AIDS. 2015;4(2):11–24. 27622004 PMC4948129

[25] Woldesenbet S , Jackson D , Lombard C , Dinh T‐H , Puren A , Sherman G , et al. Missed opportunities along the prevention of mother‐to‐child transmission services cascade in South Africa: uptake, determinants, and attributable risk (the SAPMTCTE). PLoS One. 2015;10(7):e0132425. 26147598 10.1371/journal.pone.0132425 PMC4492960

[26] Colvin CJ , Konopka S , Chalker JC , Jonas E , Albertini J , Amzel A , et al. A systematic review of health system barriers and enablers for antiretroviral therapy (ART) for HIV‐infected pregnant and postpartum women. PLoS One. 2014;9(10):e108150. 25303241 10.1371/journal.pone.0108150 PMC4193745

[27] Herlihy JM , Hamomba L , Bonawitz R , Goggin CE , Sambambi K , Mwale J , et al. Implementation and operational research: integration of PMTCT and antenatal services improves combination antiretroviral therapy uptake for HIV‐positive pregnant women in Southern Zambia: a prototype for Option B+? J Acquir Immune Defic Syndr. 2015;70(4):e123–9. 26181813 10.1097/QAI.0000000000000760 PMC6754251

[28] Hodgson I , Plummer ML , Konopka SN , Colvin CJ , Jonas E , Albertini J , et al. A systematic review of individual and contextual factors affecting ART initiation, adherence, and retention for HIV‐Infected pregnant and postpartum women. PLoS One. 2014;9(11):e111421. 25372479 10.1371/journal.pone.0111421 PMC4221025

[29] Moran NF , Moodley J . The effect of HIV infection on maternal health and mortality. Int J Gynaecol Obstet. 2012;119 Suppl. 1:S26–9. 22889550 10.1016/j.ijgo.2012.03.011

[30] Suthar AB , Vitoria MA , Nagata JM , Anglaret X , Mbori‐Ngacha D , Sued O , et al. Co‐trimoxazole prophylaxis in adults, including pregnant women, with HIV: a systematic review and meta‐analysis. Lancet HIV. 2015;2(4):e137–50. 26424674 10.1016/S2352-3018(15)00005-3

[31] Churchyard GJ , Mametja LD , Mvusi L , Ndjeka N , Hesseling AC , Reid A , et al. Tuberculosis control in South Africa: successes, challenges and recommendations. S Afr Med J. 2014;104(3 Suppl 1):244–8. 24893501 10.7196/samj.7689

[32] Uwimana J , Jackson D . Integration of tuberculosis and prevention of mother‐to‐child transmission of HIV programmes in South Africa. Int J Tuberc Lung Dis. 2013;17(10):1285–90. 24025379 10.5588/ijtld.12.0068

[33] Taylor AW , Mosimaneotsile B , Mathebula U , Mathoma A , Moathlodi R , Theebetsile I , et al. Pregnancy outcomes in HIV‐infected women receiving long‐term isoniazid prophylaxis for tuberculosis and antiretroviral therapy. Infect Dis Obstet Gynecol. 2013;2013:195637. 23533318 10.1155/2013/195637 PMC3606726

[34] Calvert C , Ronsmans C . HIV and the risk of direct obstetric complications: a systematic review and meta‐analysis. PLoS One. 2013;8(10):e74848. 24124458 10.1371/journal.pone.0074848 PMC3790789

[35] Browne JL , Schrier VJ , Grobbee DE , Peters SA , Klipstein‐Grobusch K . HIV, Antiretroviral therapy, and hypertensive disorders in pregnancy: a systematic review and meta‐analysis. J Acquir Immune Defic Syndr. 2015;70(1):91–8. 26322669 10.1097/QAI.0000000000000686

[36] Myer L , Phillips TK . Beyond “Option B+”: understanding antiretroviral therapy (ART) adherence, retention in care and engagement in ART services among pregnant and postpartum women initiating therapy in sub‐Saharan Africa. J Acquir Immune Defic Syndr. 2017;75:S115–22. 28498180 10.1097/QAI.0000000000001343

[37] Haas AD , Tenthani L , Msukwa MT , Tal K , Jahn A , Gadabu OJ , et al. Retention in care during the first 3 years of antiretroviral therapy for women in Malawi's option B+ programme: an observational cohort study. Lancet HIV. 2016;3:e175–82. 27036993 10.1016/S2352-3018(16)00008-4 PMC4904064

